# Prevalence of *Theileria* and *Babesia* species in Tunisian sheep

**DOI:** 10.4102/ojvr.v83i1.1040

**Published:** 2016-05-24

**Authors:** Mohamed R. Rjeibi, Mohamed A. Darghouth, Mohamed Gharbi

**Affiliations:** 1 Institution of Agricultural Research and Higher Education, Laboratory of Parasitology, National School of Veterinary Medicine, Manouba University, Tunisia; 2 Department of Biology, Carthage University, Tunisia

## Abstract

In this study, the prevalence of *Theileria* and *Babesia* species in sheep was assessed with Giemsa-stained blood smear examination and polymerase chain reaction to identify the different piroplasms in 270 sheep from three Tunisian bioclimatic zones (north, centre, and south). The overall infection prevalence by *Babesia* spp. and *Theileria* spp. in Giemsa-stained blood smears was 2.9% (8/270) and 4.8% (13/270) respectively. The molecular results showed that sheep were more often infected by *Theileria ovis* than *Babesia ovis* with an overall prevalence of 16.3% (44/270) and 7.8% (21/270) respectively (*p* = 0.01). The molecular prevalence by *Babesia ovis* was significantly higher in females than in males (*p* < 0.05). According to localities *B. ovis* was found exclusively in sheep from the centre of Tunisia (Kairouan) whereas *Theileria ovis* was found in all regions. Infections with *T. ovis* and *B. ovis* were confirmed by sequencing. The sequence of *T. ovis* in this study (accession numbers KM924442) falls into the same clade as *T. ovis* deposited in GenBank. The *T. ovis* amplicons (KM924442) showed 99%–100% identities with GenBank sequences. Moreover, comparison of the partial sequences of 18S rRNA gene of *B. ovis* described in this study (KP670199) revealed 99.4% similarity with *B. ovis* recently reported in northern Tunisia from sheep and goats. Three nucleotides were different at positions 73 (A/T), 417 (A/T), and 420 (G/T). It also had 99% identity with *B. ovis* from Spain, Turkey and Iraq. The results suggest a high *T. ovis* prevalence in Tunisia with a decreasing north-south gradient. This could be correlated to the vector tick distribution.

## Introduction

Small ruminant piroplasmosis is an important haemoprotozoan infection of sheep in tropical and subtropical regions (Altay, Dumanli & Aktas 2007). The piroplasms cause diseases that impair the development and productivity of the livestock industry and result in severe economic losses (Zhang *et al.* 2014). Piroplasmoses caused by *Theileria* and *Babesia* species lead to clinical infections in domestic and wild animals as well as in humans (Aydin, Aktas & Dumanli 2015). Theileriosis in small ruminants is caused by at least six species, namely *Theileria ovis*, *Theileria separata*, *Theileria recondita*, *Theileria lestoquardi*, *Theileria uilenbergi* and *Theileria luwenshuni* (Li *et al.* 2009; Schnittger *et al.* 2003; Zhang *et al.* 2014). *Theileria ovis* and *T. separata* are low- or non-pathogenic species (Friedhoff 1997), whereas *T. lestoquardi* is classified as malignant because it causes high mortality rates reaching 100% in some regions (Ahmed *et al.* 2003). In Tunisia, *T. ovis* was first reported in sheep using molecular techniques by M’ghirbi *et al*. (2013) and Rjeibi *et al.* (2014b). Recently, Rjeibi *et al.* (2014a) reported *T. lestoquardi* for the first time in sheep in southern Tunisia.

Babesiosis is a haemoparasitic disease belonging to a complex of several tick-borne diseases with different aetiological agents, such as protozoa, rickettsiae, and bacteria (Ranjbar-Bahadori *et al.* 2011) transmitted by ixodid ticks (Aktas, Altay & Dumanli 2007). The high lethality and morbidity caused by babesiosis explain its importance as a major constraint to livestock breeding development (Ahmed *et al*. 2002; Mehlhorn, Schein & Ahmed 1994). Several species of *Babesia* (*Babesia ovis*, *Babesia motasi*, *Babesia crassa,* and *Babesia* sp. Xinjiang) have been described in sheep; among them *B. ovis* and *B. motasi* are causative agents of sheep babesiosis (Liu *et al.* 2007; Ranjbar-Bahadori *et al.* 2011; Schnittger *et al.* 2003; Uilenberg 2001). In Tunisia, only *B. ovis* has been reported in small ruminants using molecular tools (Rjeibi *et al.* 2014b).

*Babesia motasi* is moderately virulent, whereas *B. crassa* appears to have little or no pathogenicity (Hashemi-Fesharki 1997). *Haemaphysalis punctata* is the vector of this species and is widespread in tropical Africa (Uilenberg *et al.* 1980). The most important *Babesia* species infecting small ruminants is *B. ovis*, which has been reported in Europe, Africa, Asia, and the Far East (Ahmed *et al.* 2006). *Babesia ovis* is highly pathogenic, especially in sheep; it causes severe infections characterised by fever, anaemia, icterus, and haemoglobinuria. Mortality rates in susceptible hosts range from 30% to 50% in natural infections (Aktas, Altay & Dumanli 2005).

The advances in molecular biology enable genotypic characterisation and have proven to be useful for the identification and classification of several haemoparasites (Caccio *et al.* 2000). These molecular techniques are highly sensitive and specific compared to Giemsa-stained and serological techniques (Papadopoulos, Brossard & Perie 1996). The aim of the present study was to study the sheep piroplasms in Tunisia using Giemsa-stained blood smears, polymerase chain reaction (PCR), and PCR–restriction fragment length polymorphism (RFLP). Genetic characterisation was performed on the Tunisian isolates.

## Materials and methods

### Study region and sample collection

**FIGURE 1:**
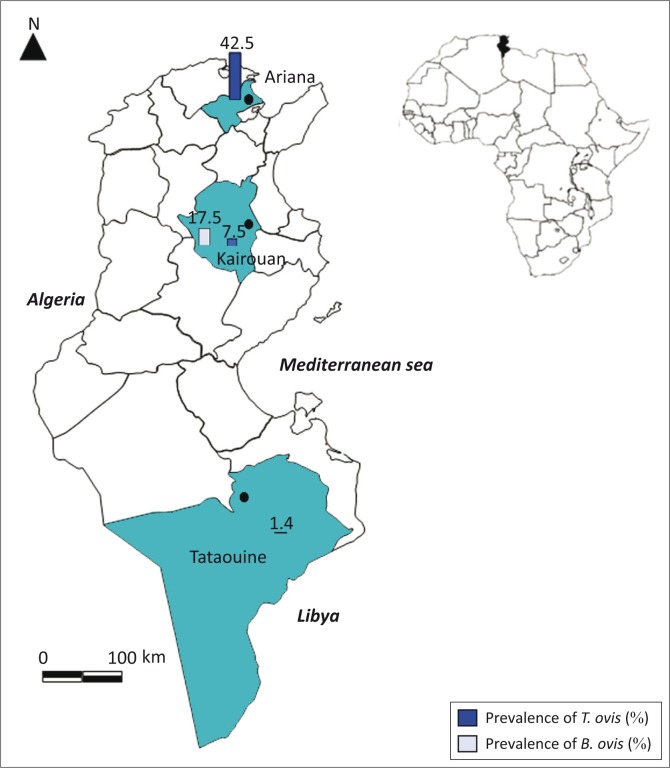
*Babesia ovis* and *Theileria ovis* molecular prevalence in studied Tunisian localities.

The present study was carried out on 270 Barbarine sheep from three traditionally managed farms located in three Tunisian governorates (semi-arid, arid and Saharan) (Figure 1). Data concerning altitude, annual rainfall, temperature, and moisture in the three study regions are reported in Table 1.

**TABLE 1:** Geographic and abiotic characteristics of the studied Tunisian sheep.

Region	Governorate	Farm sheep population	Sampled sheep	Bioclimatic zone	Mean altitude§	MAT†		MAP‡ (mm)§	Relative humidity (%): Min–Max§

						°C	Min–Max§		
North	Ariana	220	80	Semi-arid	25	18.4	7–33	450	39–92
Centre	Kairouan	400	120	Arid	68	19.5	6–37	308	28–95
South	Tataouine	150	70	Saharan	247	20.5	6–38	51	15–98

† Mean annual temperature;

‡ Mean annual precipitation;

§ Climatic data were gathered from Weather Online (n.d.) and Climatedata.eu. (n.d.).

Based on their dentition, sheep were ranked into two age groups: less than and more than 2 years of age.

Blood samples were collected in EDTA tubes from each animal and stored at -20 °C until used. Giemsa-stained blood smears were examined microscopically with immersion oil at 1000x magnification for the presence of piroplasms. All the sheep included in the present survey were examined for ticks, which were collected and placed in tubes with 70% ethanol, then identified using the key of Walker *et al*. (2003). Three tick infestation indicators were determined (Margolis *et al*. 1982):

- Infestation prevalence (%) = 100 × (number of infested sheep/total number of sheep).
- Infestation intensity = number of ticks/number of infested sheep.
- Abundance = number of ticks/total number of sheep.

#### *Theileria* spp. polymerase chain reaction and polymerase chain reaction–restriction fragment length polymorphism

DNA was extracted from 300 μL of whole blood with the Wizard^®^ Genomic DNA purification kit (Promega, Madison, USA) according to the manufacturer’s instructions. DNA was stored at -20 °C until used. A nested PCR detecting specific *Theileria* DNA of the 18S rRNA gene was performed using Thei F1 and Thei R2 primers for the primary PCR and Thei F2, Thei R2 primers for the secondary PCR (Heidarpour Bami *et al.* 2009) (Table 2). Distilled water and *T. ovis* DNA (Rjeibi *et al*. 2014b) were used as negative and positive controls respectively. PCR products were separated in 1% agarose gels to check the size of the amplicons.

**TABLE 2:** 18S rRNA gene primers used for *Babesia ovis* PCR detection, semi-nested PCR of *Babesia motasi* and nested PCR of *Theileria* spp. from sheep in the present study.

Primer specificity	Name	Primers 5’-3’	Product size (bp)	Reference
*Babesia ovis*	Bbo-F	TGGGCAGGACCTTGGTTCTTCT	549	Aktas *et al*. (2005)
	Bbo-R	CCGCGTAGCGCCGGCTAAATA	-	-
*Babesia motasi*	P1	CACAGGGAGGTAGTGACAAG	389–402	Shayan *et al*. (2008)
	P2	AAGAATTTCACCTATGACAG	-	-
	P2	AAGAATTTCACCTATGACAG	205	-
	P4	CGCGATTCCGTTATTGGAG		
*Theileria* spp.	Thei F1	AACCTGGTTGAT CCTGCCAG	1700	Heidarpour Bami *et al*. (2009)
	Thei R1	AAACCTTGTTACGACTTCTC	-	**-**
	Thei F2	TGATGTTCGTTTYTACATGG	1417–1426	**-**
	Thei R2	CTAGGCATTCCTCGTTCACG	-	**-**

*Note:* Please see the full reference list of the article, Rjeibi, M.R., Darghouth, M.A. & Gharbi, M., 2016, ‘Prevalence of *Theileria* and *Babesia* species in Tunisian sheep’, *Onderstepoort Journal of Veterinary Research* 83(1), a1040. http://dx.doi.org/10.4102/ojvr.v83i1.1040, for more information.

The discrimination of three *Theileria* species (*Theileria annulata*, *T. lestoquardi*, and *T. ovis*) was done by RFLP analysis of amplicons with restriction enzyme: *HpaII* (Fermentas, Lithuania) (Heidarpour Bami *et al.* 2009). The enzymatic digestion mixture consisting of 10 μL PCR amplicons, 10x buffer, and restriction enzyme (10 U) was then incubated at 37 °C for 2 h. Restriction digests were separated by electrophoresis in 2% agarose and visualised in a UV transilluminator.

#### *Babesia ovis* polymerase chain reaction

Five microlitres of DNA of each sample were amplified by 35 PCR cycles using *B. ovis* specific primers (Bbo-F and Bbo-R) (Aktas *et al.* 2005). PCR products were separated in 1% agarose gels to check the size of amplicons (Table 2).

#### Semi-nested polymerase chain reaction detecting *Babesia motasi*

This PCR was performed in 100 μL total volume consisting of 1x PCR buffer, 2.5 U Taq polymerase (Biobasic, Canada), 2 μL of each primer P1/P2 (20 mM), 200 μM each of dNTP, and 1.5 mM MgCl_2_. Five microlitres of DNA of each sample were amplified by 38 PCR cycles in an automated thermocycler (ESCO Swift MaxPro) (Shayan *et al.* 2008). The PCR products were analysed on 1.8% agarose gel and visualised using ethidium bromide with a UV transilluminator. For specific detection of *B. motasi* DNA, semi-nested PCR was used using a species-specific primer (P4) designated within the V4 hypervariable region of 18S rRNA gene. Semi-nested PCR was performed with the purified PCR product. One microlitre of the purified PCR product was amplified with the primers P4/P2 (Shayan *et al.* 2008). Semi-nested PCR was separately performed directly with 1 μL PCR product as well. PCR products were separated in 1.8% agarose gels to check the size of the amplicons (Table 2).

### DNA sequencing and phylogenetic analysis

Three selected *T. ovis* amplicons from each region and two PCR products of *B. ovis* from Kairouan (central Tunisia) were purified with the Wizard SV gel and PCR clean-up system (Promega, Madison, USA) according to the manufacturer’s instructions. Purified DNA fragments were sequenced in both directions, using the same primers as for PCR. The reactions were performed using a conventional Big Dye Terminator cycle sequencing ready reaction kit (Applied Biosystems, Foster City, CA, USA) with an ABI3730XL automated DNA sequence.

**FIGURE 2:**
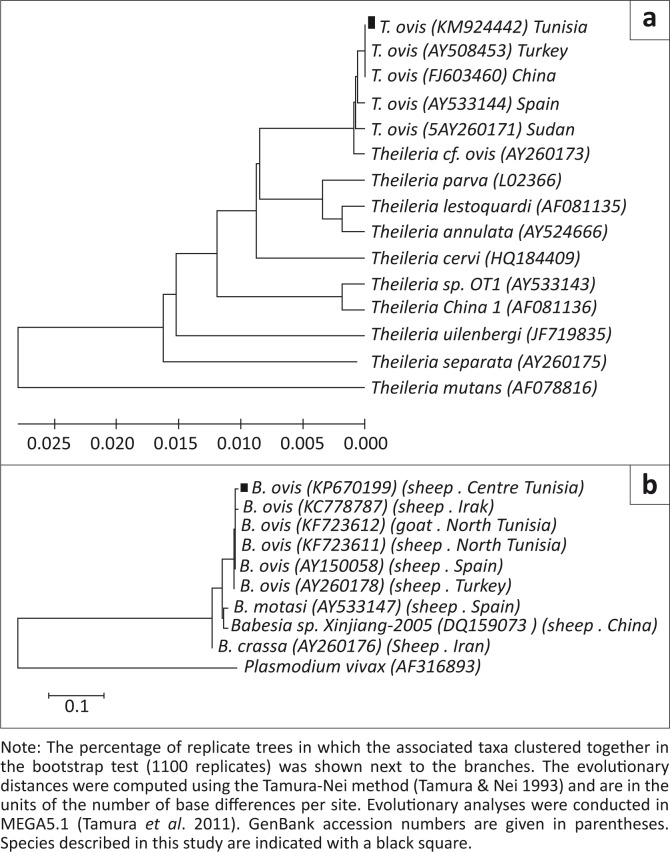
The tree was constructed using the neighbour-joining method (Saitou & Nei 1987); (a), Partial sequence 18S rRNA gene phylogenetic tree of the species identified in this survey and the main small ruminants’ *Theileria* species; (b) Partial sequence 18S rRNA gene phylogenetic tree of the species identified in this survey and the main small ruminants’ *Babesia* species.

The obtained 18S rRNA gene sequences were edited using the ChromasPro software (version 1.7.4). The pairwise nucleotide per cent identity of the new sequences was calculated using MEGA 5.1 software (Tamura *et al.* 2011). Phylogenetic trees were constructed by the neighbour-joining method (Saitou & Nei 1987). Distances were estimated by the Tamura-Nei method (Tamura & Nei 1993). The sequences of *B*. *ovis* and *T. ovis* 18S rRNA genes identified in the present survey were deposited in GenBank under accession numbers KP670199 and KM924442 respectively (Figure 2).

### Statistical analyses

The infection prevalence percentages were compared using EpiInfo6 (Dean *et al.* 2011). The concordance between PCR and blood smears was estimated with Kappa test (Toma *et al.* 2007). A probability of 0.05 was used as a threshold for statistical significance.

## Ethical considerations

This study was conducted in accordance with relevant national and international guidelines on handling animals, taking care to respect animal welfare. Agreement was obtained from all farmers before their sheep were sampled.

## Results

### Ticks population

In total 135 adult ticks were collected from 270 sheep (85 males and 50 females). The ticks’ sex ratio (M: F) was 1.7. These ticks were identified as *Hyalomma dromedarii* (28.9%) and *Rhipicephalus turanicus* (71.1%) (*p* < 0.001). The highest infestation prevalence was observed for *H. Dromedarii* (25.7%) compared to *R. turanicus* (22.5%). The overall infestation intensity and abundance were 2.14 and 0.5 ticks/animal respectively. No significant difference was observed in infestation intensity of *R. turanicus* (2.13 ticks/animal) and *H. dromedarii* (2.16) (*p* < 0.05). However, the abundance of *R. turanicus* (0.35) was significantly higher than the abundance of *H. dromedarii* (0.14) (*p* < 0.001). *H. dromedarii* was exclusively collected from south Tunisia whereas *R. turanicus* was present in both north and centre. The prevalence infestation by *R. turanicus* was significantly higher in north than in central Tunisia (*p* < 0.05).

The overall prevalence of tick infestation was 23.3% (63/270), with an intensity of 2.14 and an abundance of 0.5. The prevalence of piroplasms was not significantly different in tick-infested and non-infested sheep (*p* > 0.05).

#### *Theileria ovis* infection

The overall infection prevalence by *Theileria* spp. in blood smears was 4.8% (13/270) and the overall mean parasitaemia was 0.016% (range: 0.01% – 0.03%). The enzymatic digestion profile by *HpaII* restriction enzyme showed that all PCR positive amplicons belonged to *T. ovis* (44/270). There was no concordance between PCR and blood smears for *Theileria* spp. in sheep (*k* = 0.00).

The overall *T. ovis* prevalence was 16.29% with a north–south axis decreasing gradient. The highest prevalence was observed in Ariana (north) (42.5% ± 0.108), followed by Kairouan (centre) (7.5% ± 0.047), and Tataouine (south) (1.43% ± 0.014) (*p* < 0.001). The prevalence was higher in adult sheep (23%) compared to lambs (12.35%) (*p* = 0.02). There was no difference between prevalence rates in males and females ( *p* > 0.05) (Table 3).

**TABLE 3:** Association between *Babesia ovis* and *Theileria ovis* molecular prevalence in sheep and different parameters.

Parameter	*Babesia ovis* +ive/examined	%	*Theileria ovis* +ive/examined	%
**Gender**	-	-	-	-
Female	19/175	10.8	29/175	16.6
Male	2/95	2.1	15/95	15.8
**Age group**	-	-	-	-
< 1 year	21/170	12.3**	21/170	12.35
> 1 years	0/100	0	23/100	23*
**Locality**	-	-	-	-
North	0/80	0	34/80	42.5**
Centre	21/120	17.5**	9/120	7.5
South	0/70	0	1/70	1.4
**Overall**	**21/270**	**7.8**	**44/270**	16.3*

* 0.001 ≤ *p* < 0.05;

** *p* < 0.001

The three *T. ovis* amplicons showed 100% identity between them (830 bp length); one of them was submitted to GenBank (accession number: KM924442). The present sequence showed identities of 99% – 100% with GenBank published *T. ovis* sequences (Figure 2a). The sequence of *T. ovis* in this study falls into the same clade with all the *T. ovis* sequences from Africa, Europe, and Asia and is clearly distinct from other *Theileria* species such as *T. uilenbergi* (JF719835), *T. lestoquardi* (AF0811335), and *T. annulata* (AY524666).

#### *Babesia ovis* infection

The overall infection prevalence by *Babesia* spp. in blood smears was 2.9% (8/270) and the overall mean parasitaemia was 0.024% (range: 0.01% – 0.03%). Eight sheep were exclusively infected by *Babesia* spp. in blood smears; 21 were positive for *B. ovis* by PCR. There was no concordance between PCR and blood smears for *B. ovis* infection (*k* = 0.00). All positive blood smears were positive by *B. ovis* PCR while *B. motasi* DNA was not detected.

The overall *B. ovis* molecular prevalence was 7.8% (21/270); it was significantly higher in yearling sheep compared to adults (*p* < 0.001). The infection by *B. ovis* was found exclusively in central Tunisia (Kairouan) with a prevalence of 17.5% ± 0.06 (21/120) (*p* < 0.001). The infection rate by *B. ovis* was significantly higher in females than males (*p* = 0.01). Two *B. ovis* amplicons from Kairouan were randomly chosen for genetic analysis. The comparison of the 18S rRNA *B. ovis* sequence (509 bp length) revealed 100% identity between them; one of them has been deposited in GenBank under accession nos. KP670199. *Babesia ovis* sequences described in this study clustered with the other *B. ovis* sequences clearly distinct from *B. motasi*, *B. crassa* and *Babesia* sp. Xinjiang-2005 (Figure 2b).

It shared 99.4% identity with a *B. ovis* 18S rRNA sequence reported recently in northern Tunisia from sheep and goats respectively (KF723611 and KF723612). Three nucleotides were different at positions 73 (A/T), 417 (A/T), and 420 (G/T). The present sequences showed 99.4%, 99.2% and 99% identity with *B. ovis* from Spain (AY150058), Turkey (AY260178), and Iraq (KC778787), respectively (Figure 2b).

## Discussion

Piroplasmoses are caused by the tick-borne haemoprotozoans *Theileria* and *Babesia* and have a major impact on livestock production in tropical and subtropical regions (Altay *et al.* 2007). In the present study, we estimated the prevalence and distribution of *Theileria* and *Babesia* species in Tunisian sheep in three regions (north, centre, and south). We detected *Theileria* spp. and *Babesia* spp. in blood smears of 4.8% and 2.9% sheep respectively, whereas the molecular prevalence of *T. ovis* and *B. ovis* was significantly higher (16.3% and 7.8% respectively) (*p* < 0.05). This difference shows that the PCR is a reliable screening method in carrier sheep. Indeed, the parasitaemia in the present study was very low (range: 0.01% – 0.03%). This is in agreement with other studies in Greece (Papadopoulos *et al.* 1996), Turkey (Razmi *et al.* 2003) (0.01% – 0.1%), and recently in northern Tunisia (Rjeibi *et al.* 2014b) (0.01% – 0.05%). Our research showed that sheep were more frequently infected by *T. ovis* (16.3%) than *B. ovis* (7.8%) (*p* < 0.05). This in agreement with the findings of Nagore *et al*. (2004) who found that sheep were more often infected by *T. ovis* (18%) than *B. ovis* (2.5%), contrary to Rjeibi *et al.* (2014b) who showed that sheep were more infected by *B. ovis* (17.4%) than *T. ovis* (5.8%).

Concerning *Theileria* species, our survey showed that only *T. ovis* was present in Tunisian sheep, this in agreement with M’ghirbi *et al*. (2013) and Rjeibi *et al.* (2014b). The same results were reported in Turkey (Altay *et al.* 2007). In the present study, no *T. lestoquardi* was isolated, but Rjeibi *et al.* (2014a) detected two positive animals, raising the question whether this species is established in Tunisia or not.

There is a decreasing north-south prevalence gradient; indeed, *T. ovis* was highly prevalent in Ariana (north) (42.5%) compared to Tataouine (south) (1.43%) (*p* < 0.001). This gradient was also in accordance with *R. turanicus* distribution, which was more prevalent in Ariana (46.25%) followed by Kairouan (6.66%), whereas this tick species was absent in the south (*p* < 0.001). These observations strongly suggest that in Tunisia *R. turanicus* is the vector of *T. ovis.*

Regarding *Babesia* species, no *B. motasi* was detected in this study; sheep were exclusively infected by *B. ovis* with an overall infection prevalence of 7.8% (21/270). Similar results were reported in Turkey and Iran where small ruminants were only infected by *B. ovis* (Altay *et al.* 2007; Esmaeilnejad *et al.* 2014). In northern Spain, *B. ovis* and *B. motasi* were present in sheep with an overall prevalence of 2.5% and 2% respectively (Nagore *et al.* 2004). *Babesia motasi* is transmitted by *Haemaphysalis* spp. (Uilenberg 2006); this species and *B. ovis* are the primary agents of ovine babesiosis. Geographic *B. motasi* isolates have different virulence. For example, in northern Europe it has low pathogenicity whereas in the Mediterranean Basin this species is highly pathogenic (Uilenberg 2006).

The highest *B. ovis* infection rate was observed in female sheep (10.8%) compared to males (2.1%) (*p* < 0.05). This is contradictory to the recent survey in northern Tunisia that reported no difference in *B. ovis* prevalence between males and females (Rjeibi *et al.* 2014b). Iqbal *et al*. (2011) in Pakistan showed that males were more infected than females. This could be explained by the fact that the majority of female sheep graze whereas males are kept indoors.

Yearling sheep were exclusively infected by *B. ovis* with a prevalence of (10.8%) (*p* < 0.001); our results are consistent with those reported in Pakistan where the prevalence in animals aged less than 1 year was higher (Iqbal *et al.* 2011). These results suggest that the visited farms are in a state of enzootic stability for *B. ovis* infection, contrary to the findings in Turkey and Iran (Aktas *et al.* 2007; Razmi *et al.* 2002, 2003) where no significant difference between animals’ ages and *B. ovis* infection prevalence was reported.

Overall prevalence rates of *B. ovis* differed statistically among localities (*p* > 0.001); it was only observed in Kairouan (central Tunisia), with a prevalence of 17.5%, where sheep were only infested by *R. turanicus*, which is the potential vector of *B. ovis* (Table 3). *Rhipicephalus turanicus* was reported by Bouattour (1987) in the northern and central regions of Tunisia but no specimens were collected in the south. On the contrary, Rjeibi *et al.* (2014b) reported that sheep were infected by *B. ovis* in northern Tunisia with a prevalence of 17.4% (30/172).

There was no association between tick burdens and piroplasm prevalence (*p* > 0.05). This indicator has little value because this study was trans-sectional and carried out on healthy animals. Other findings reported the presence of a positive correlation between tick burdens and infection prevalence (Aktas *et al.* 2005; Durrani *et al.* 2012; Iqbal *et al.* 2013).

The Turkish and Chinese isolates (AY508453 and FJ603460) had 100% identity with our sequences. All the *T. ovis* sequences from Africa, Europe, and Asia clustered together in a single clade that was divergent from other ruminants’ pathogenic *Theileria*, namely *T. uilenbergi* (JF719835), *T. lestoquardi* (AF0811335), and *T. annulata* (AY524666). Our *T. ovis* amplicons (830 bp length) showed 100% identity with recently reported Tunisian *T. ovis* isolated (Rjeibi *et al.* 2014b) (Figure 2a).

The *B. ovis* sequence described in this study from central Tunisia (KP670199) had 99.4% similarity with the recently reported sequence in northern Tunisia (KF723612) (Rjeibi *et al.* 2014b).This could be explained by the absence of genetic mixing between the two parasite populations.

In the phylogenetic tree, our samples clustered with all *B. ovis* in a single clade. Phylogenetic analyses provided evidence that *B. ovis* is distinct from other *Babesia* species, namely, *B. motasi*, *B. crassa* and *Babesia* sp. Xinjiang-2005.

These results suggest a high *T. ovis* prevalence in Tunisia with a decreasing north-south gradient corresponding to geographic distribution *R. turanicus*.

## Conclusion

Further studies on representative tick samples are needed to establish the list of tick vectors of sheep piroplasms in Tunisia. M’ghirbi *et al*. (2013) found neither *B. ovis* nor *T. ovis* DNA in *Rhipicephalus bursa* (*N* = 10) and *R. turanicus* (*N* = 215). The pathogenicity of *T. ovis* either alone or in association with other pathogens is to be investigated.
